# A Bionic Testbed for Cardiac Ablation Tools

**DOI:** 10.3390/ijms232214444

**Published:** 2022-11-21

**Authors:** Wei-Han Lin, Zhijie Zhu, Vasanth Ravikumar, Vinod Sharma, Elena G. Tolkacheva, Michael C. McAlpine, Brenda M. Ogle

**Affiliations:** 1 Department of Biomedical Engineering, University of Minnesota—Twin Cities, Minneapolis, MN 55455, USA; 2 Stem Cell Institute, University of Minnesota—Twin Cities, Minneapolis, MN 55455, USA; 3 Department of Mechanical Engineering, University of Minnesota—Twin Cities, Minneapolis, MN 55455, USA; 4 Department of Electrical Engineering, University of Minnesota—Twin Cities, Minneapolis, MN 55455, USA; 5 Cardiac Rhythm and Heart Failure Division, Medtronic Inc., Minneapolis, MN 55432, USA; 6 Lillehei Heart Institute, University of Minnesota—Twin Cities, Minneapolis, MN 55455, USA; 7 Institute for Engineering in Medicine, University of Minnesota—Twin Cities, Minneapolis, MN 55455, USA; 8 Masonic Cancer Center, University of Minnesota—Twin Cities, Minneapolis, MN 55455, USA

**Keywords:** 3D printing, bioprinting, cryoablation, medical device testbeds, induced pluripotent stem cells, tissue engineering, soft sensors

## Abstract

Bionic-engineered tissues have been proposed for testing the performance of cardiovascular medical devices and predicting clinical outcomes ex vivo. Progress has been made in the development of compliant electronics that are capable of monitoring treatment parameters and being coupled to engineered tissues; however, the scale of most engineered tissues is too small to accommodate the size of clinical-grade medical devices. Here, we show substantial progress toward bionic tissues for evaluating cardiac ablation tools by generating a centimeter-scale human cardiac disk and coupling it to a hydrogel-based soft-pressure sensor. The cardiac tissue with contiguous electromechanical function was made possible by our recently established method to 3D bioprint human pluripotent stem cells in an extracellular matrix-based bioink that allows for in situ cell expansion prior to cardiac differentiation. The pressure sensor described here utilized electrical impedance tomography to enable the real-time spatiotemporal mapping of pressure distribution. A cryoablation tip catheter was applied to the composite bionic tissues with varied pressure. We found a close correlation between the cell response to ablation and the applied pressure. Under some conditions, cardiomyocytes could survive in the ablated region with more rounded morphology compared to the unablated controls, and connectivity was disrupted. This is the first known functional characterization of living human cardiomyocytes following an ablation procedure that suggests several mechanisms by which arrhythmia might redevelop following an ablation. Thus, bionic-engineered testbeds of this type can be indicators of tissue health and function and provide unique insight into human cell responses to ablative interventions.

## 1. Introduction

Cardiac tissue engineering has shown promise for generating in vitro living tissue model systems for studying diseases, as well as in vivo therapeutics to cure diseases. Many studies have demonstrated that cardiomyocytes can be deposited with or without biocompatible scaffolds and shaped into simple structures such as spheroids [1], strips [2], and cups [3]. These tissues can serve as platforms to study healthy and diseased human models and to predict the clinical outcomes of pharmaceutical drugs [4,5,6]. Cardiac tissue engineering also has potential to aid in the evaluation and rehearsal of cardiac surgical procedures. The ideal engineered cardiac tissue would be large enough to accommodate a medical device (i.e., of at least centimeter scale) and would harbor a response element for the real-time monitoring of environmental conditions—a mainstay for FDA approval processes.

**Figure 1:**
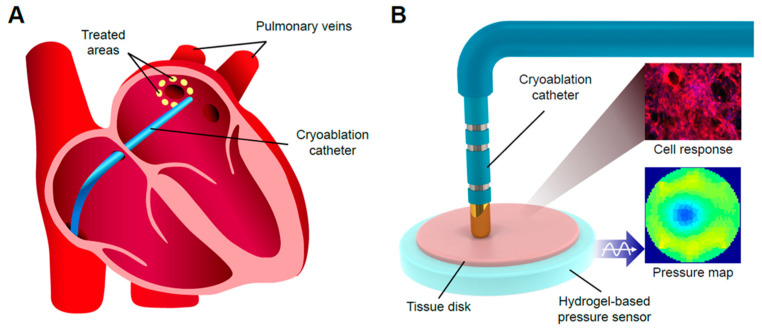
(**A**) Schematic image showing the process of pulmonary vein ablation (two of four veins shown). (**B**) Schematic image showing the bionic myocardial testbed with pressure-sensing capability.

In this manuscript, we explore the case of cardiac ablation therapy through left atrial pulmonary vein ablation, which is an effective way to treat cardiac arrhythmias, including atrial fibrillation (AF), which is common among aging populations [7,8]. To achieve pulmonary vein isolation during ablation, a catheter tip is inserted into the left atrium, applying or removing energy to the tissue surrounding it, and presumably creating a lesion area to block the entry of abnormal signals from the pulmonary veins (Figure 1A).

In an ablation process, contact force is critical to lesion area formation [9,10]. Contact forces that are too high, too low, or applied non-uniformly can lead to complications. Abnormal contact force can induce cardiac perforation and tamponade during radio frequency ablation treatment [11,12,13] and chest pain during cryocatheter ablation treatment [14]. As a potential consequence of low contact forces, the recurrence of AF is common after cardiac ablation therapy and is thought to reflect pulmonary vein reconnection. In the long term, 70–75% of patients suffer recurrent AF within 5 years [15,16]. Rare but fatal complications including pulmonary vein stenosis, atrioesophageal fistula, and nerve injury have also been reported [17], and these may also be related to suboptimal contact force. Ablation therapy is also commonly used to treat AV node reentrant tachycardia (AVNRT) and has the similar issue that low contact force can result in ineffective tissue ablation and AVNRT recurrence. The 6-month post-ablation recurrence rate for AVNRT is 9.4% after treatment with cryoablation [18].

To examine novel ablation tools under preclinical development and to monitor process parameters including contact force, animal tissues and polymeric soft materials have been adopted in ablation testbeds as tissue phantoms [11,13,19,20]. These models exhibited several limitations, including inherent differences compared to human cardiac electrophysiology [21] and variations in technical approaches [22]. While in silico models are useful for comparing ablation techniques [23] and providing electrical functioning information to identify ablation targets [24], the translational value of these models is limited by a need for further biological validation reflective of patient electrophysiology. Therefore, the inclusion of living human cardiac muscle in a device testing platform with sensing modules will be important for the prediction of outcomes in patients.

To track pressure changes during catheter intervention, the pressure sensing element is indispensable for a testbed. A common method of pressure sensing on a tissue testbed is to integrate pressure sensing modules onto the catheter used in the course of the ablation procedure [25,26]. Major drawbacks of this approach include: (1) additional complexity to adapt the pressure sensors to the catheters’ compact design; and (2) the requirement of spatial sensing modules, such as cameras, to record the locations of ablation sites. Cameras may encounter ambiguous visual features at ablation sites, presenting additional challenges. Rather than integrating sensing functions into the catheters, we developed a soft hydrogel sensor to be integrated with the engineered living cardiac tissue (Figure 1B).

Our sensor design is based on electrical impedance tomography (EIT), which can provide a desirable spatial resolution of pressure mapping while maintaining a simple geometric design. An EIT sensor consists of a continuous piece of conductive material and electrodes distributed along the boundary. The application of pressure on the conductive material can induce deformation and thus a variation in conductance, the distribution of which can be mapped based on electrical measurements from different pairs of electrodes [27]. Conductive fabric [28,29] and carbon–elastomer composites [30,31,32] have been used in EIT sensors for pressure sensing. The sensitivity of the conductive fabric is too low for the detection of small forces applied during ablation, with the minimum detectable force of ~100 g [32] being one order of magnitude higher than the typical range of contact force for the ablation of ~5–15 g [33]. Carbon–elastomer sensors, on the other hand, are not accurate for real-time continuous recording due to irreversible responses and long settling times [30]. These issues can be resolved by adopting an ionic hydrogel as the conductive material. Ionic hydrogels possess tissue-like softness and high transparency, and exhibit variable conductance in response to different levels of strain [34]. Recently, we demonstrated the capability of a hydrogel-based EIT sensor for strain mapping [35]. In this work, we extended the function of the hydrogel sensor for pressure sensing. The sensor design was optimized for biocompatibility with the cardiac cells. The sensor was operated in saline solution to mimic the in vivo ablation environment. A low-cost, casting-based fabrication process aided by 3D-printed molds was also developed for the scalable manufacturing of the sensor.

To accommodate the catheter size, centimeter-scale human cardiac tissues are needed. However, creating these kinds of tissues in vitro with contiguous cell layers and functional cell-to-cell coupling has been challenging, given that cardiomyocytes display limited capacity for proliferation and migration. We overcame this obstacle by adopting a strategy that allows human-induced pluripotent stem cells (hiPSCs) to proliferate to high densities in the three-dimensional (3D) construct; then, we differentiated them into cardiomyocytes in situ [36]. This was accomplished by developing an extracellular matrix-based bioink optimized for hiPSC expansion and cardiac differentiation [37].

The resulting bionic testbed combines the hydrogel sensor with the centimeter-scale tissue to perform the spatiotemporal recording of pressure distributions over a tissue disk during ablation tests. Here, we evaluate for the first time the extent to which applied pressure during ablation alters the treated area and cell responses in the affected area of living human cardiac tissue.

## 2. Results

### 2.1. Hydrogel-Based EIT Pressure Sensor

#### 2.1.1. Sensor Fabrication

**Figure 2:**
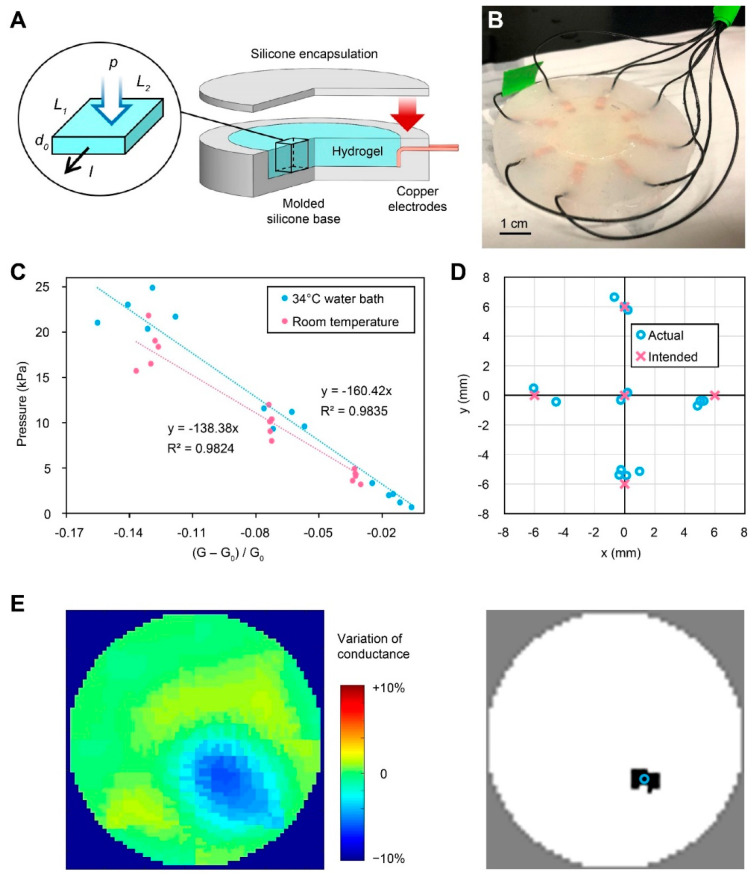
(**A**) Schematic image showing the layered design of the hydrogel-based pressure sensor with the inset image showing an infinitesimal region within the hydrogel layer. (**B**) Photograph of the fabricated hydrogel-based pressure sensor. (**C**) Plot showing the curves to predict the applied pressure based on inputs from a hydrogel sensor. The linear curves are fitted from the calibration dataset consisting of applied pressure (measured with the force gauge) and the variation in conductance (measured with the hydrogel sensor) in a 34 °C water bath and under room temperature (*n* = 15 readings from at least 3 different hydrogel sensors). G denotes conductance under pressure, G_0_ denotes initial conductance without pressure. (**D**) Plot showing the detected and intended positions of point-wise compression at multiple locations on the sensor in 34 °C water bath (*n* = 20). (**E**) Images showing the map of variation in conductance defined by (G − G_0_)/G_0_ (left), and the estimated pressing region (in black) and catheter location (blue circle) within the circular sensing region.

The soft sensor consists of a soft silicone substrate, an ionic hydrogel layer for pressure sensing, copper electrodes at the boundary for current injection and voltage measurement, and a top silicone layer for encapsulation to enable operation in saline solution (Figure 2A). Lithium chloride was selected to provide mobile ions in the hydrogel matrix because of its hygroscopic property, which helps to prevent dehydration during fabrication. The Young’s modulus of the ionic hydrogel was ~16 kPa at 50% compressive strain (Figure S1), which is on the same scale as the Young’s modulus of cardiac tissue [38]. Matching the Young’s modulus enhanced the mechanical compliance of the top tissue disk to the bottom hydrogel sensor under compressive strain, allowing the tissue disk to deform naturally under ablation catheter compression. A platinum cure silicone rubber (Ecoflex 00-30, Smooth-On, Inc., Macungie, PA, USA) was adopted in the top silicone layer and the silicone substrate, which was hydrophobic and served as the encapsulation layer to prevent current leakage to the saline solution during sensor operation. The silicone material also served as an interface with the tissue disk attached on top of the hydrogel sensor, which was confirmed to be biocompatible via cell viability tests (Figure S2). During the vertical compression of the ablation catheter, the high aspect ratio of the top silicone layer (~1 mm thickness, 30 mm diameter) reduced the impact of its higher compressive modulus (Figure S1) to the soft hydrogel layer underneath.

A customized casting process was developed for sensor fabrication (Figure S3). This enabled the rapid parallel fabrication of multiple devices with repeatable performances. First, silicone substrates with circular wells (4 mm deep, 30 mm diameter) were cast on 3D-printed plastic molds. Eight copper electrodes were embedded in each silicone substrate and were evenly distributed around the circular wells, enabled by the fixtures in the plastic molds. The copper electrodes were soldered to rubber-coated conductive wires and then encapsulated in silicone to become waterproof. Next, the hydrogel precursor was cast in the well of the silicone substrate to form the circular-shaped conductive layer. After the ionic hydrogel layer was photo-crosslinked under UV light (OmniCure Model S1500, Excelitas Technologies Corp., Waltham, MA, USA), a top layer of silicone was drop-cast on the ionic hydrogel to seal the conductive layer (Figure 2B).

#### 2.1.2. Electromechanical Characterization

During EIT operation, two pairs of electrodes at a time were selected for current sourcing and voltage measurement to perform a four-probe impedance measurement. After taking such impedance measurements from all permutations of adjacent electrode pairs, the conductance distribution within the hydrogel layer can be estimated by solving the inverse problem of a finite element model [27]. The design of the peripheral circuitry that facilitated this EIT operation has been reported in previous studies [35]. The EIT procedure was performed with a refreshing frequency of 2.5 Hz to enable the real-time reconstruction of the conductance map. To correlate these data with the pressure map, we developed a model that established the correspondence between material conductance and the applied vertical pressure.

For an infinitesimal region of the conductive layer in a rectangular shape (Figure 2A), let $L_{1}$ denote the length of the material along the direction of current flow, $L_{2}$ denote the width of the material, and $d_{0}$ denote the thickness of the material. The bulk conductivity $\sigma_{0}$ of ionic hydrogel was assumed to be constant in the model, because it has been reported that the conductivity of ionic hydrogel is independent of deformation [34,39]. Based on Pouillet’s law, a hypothesis of a linear relationship between ε, the compressive strain in thickness direction, and Δk, the change in material conductance under pressure, is expressed in Equation (1).

(1) $$\Delta k=\sigma_{0}\frac{\varepsilon L_{2}d_{0}}{L_{1}}.\;$$

During small compressive strain (<50%), the material was expected to deform within the linear region of the stress–strain curve of the hydrogel (Figure S1). Thus, the applied pressure p and the resulting compressive strain ε should follow Equation (2), with E denoting the approximated Young’s modulus in the linear compression region.

(2) $$p=E\varepsilon =\frac{EL_{1}}{\sigma_{0}L_{2}d_{0}}\Delta k.$$

The hypothesis of this linear relationship between pressure p and variation in conductance Δk was verified by measuring the sensor responses when different known levels of force were applied to the sensor via a custom-built actuator. Specifically, the 3D motion of a force probe (Torbal FB5) with a cylindrical presser (3 mm diameter, same as the dimension of the cryoablation catheter) was driven by a 3D printing gantry system (AGS 100, Aerotech Inc., Pittsburgh, PA, USA). The force probe measured the magnitude of vertical force applied to different locations on the hydrogel sensor under different vertical displacements of the presser (Figure S4). The hydrogel sensors were tested in a 34~35 °C water bath to mimic the ablation environment. Note that the conductivity of the hydrogel material was dependent on temperature, which could affect the precision of the pressure measurement. Thus, a calibration was performed after the conductance reached the equilibrium state, which took approximately 10 min after the sensor was placed in a water bath (Figure S5).

The experimental result of the point-wise compression of the hydrogel sensor under the 34 °C water bath indicated a linear relationship between the reduction in material conductance and the applied pressure (R^2^ = 0.9835, *n* = 15). This result agreed with the hypothesis of the linear model (Figure 2C). The linear ratio between the pressure and the variation in conductance can then be incorporated in a customized EIT algorithm for real-time pressure mapping during tissue cryoablation tests. A linear response of the sensor was also observed in a similar experiment conducted under room temperature without a water bath, which demonstrated the robustness of the pressure sensor under different temperatures. Note that, compared to the results under a water bath condition, in air and at room temperature, a lower pressure on the sensor yielded the same level of reduction in conductance. One hypothesis is that additional pressing force was required to overcome the buoyant force on the presser and the sensor when submerged in water.

Spatial sensing accuracy was evaluated by first extracting the coordinate of the pressing location in each reconstructed conductance map, and then comparing it with the intended pressing location executed by the gantry system (Figure 2D,E, Video S1). The spatial sensing error was 0.77 ± 0.49 mm (*n* = 20), which was sufficient for locating the ablation sites and the evaluation of ablation quality.

### 2.2. Bioprinted Centimeter-Scale Human Cardiac Disk

To create a bionic myocardial testbed for medical devices, engineered cardiac tissues with sizable dimensions compatible to the device are needed. Most macroscale engineered cardiac tissues are either created with animal cells [40] or human stem-cell-derived cardiomyocytes [41]. While the latter present human cardiac physiology more precisely, a high cell density is challenging to achieve given the low migratory and proliferative capacity of cardiomyocytes. In our previous work, an alternative approach was adopted: hiPSCs were directly embedded into extracellular matrix-based bioink solution to form crosslinked constructs, and cardiac differentiation was induced following cell expansion to yield contiguous muscularization and electrochemical connectivity. To accommodate the low viscosity of the optimized bioink solution, the freeform reversible embedding of the suspended hydrogels (FRESH) method [42] was applied to allow the 3D printing of simple (e.g., disks used here) or complex (i.e., chambered muscle pumps) structures [37].

Disks 3 cm in diameter and 1 mm in thickness were used as the cardiac tissue model to fully accommodate the cryoablation tip catheter Freezor MAX (tip diameter 3 mm). Thus, the 3D printing of the disk model allowed flat and defined disk surfaces to be generated and the hydrogel-based EIT sensors to be well integrated.

#### 2.2.1. Cardiac Disk Preparation

**Figure 3:**
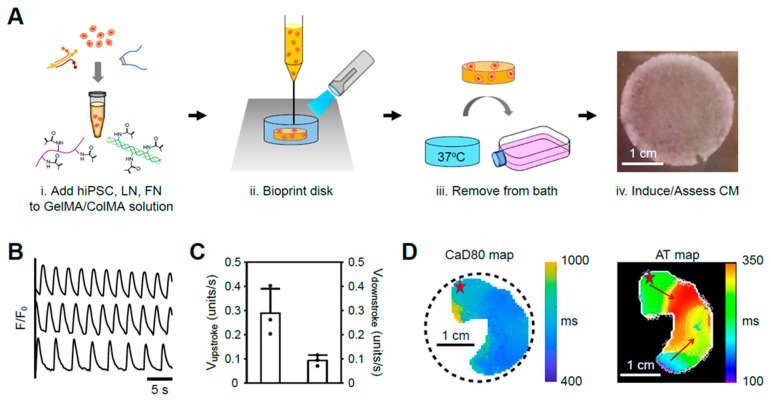
Cardiac disk preparation and characterization. (**A**) Cardiac disks were bioprinted with the bioink composed of human-induced pluripotent cells (hiPSCs) and extracellular proteins. The inset in subfigure iv represents a differentiated cellular disk with a scalpel-made marker for structure orientation. CM indicates cardiomyocyte; ColMA, collagen methacrylate; FN, fibronectin; GelMA, gelatin methacrylate; and LN, laminin-111. (**B**,**C**) Calcium traces generated from differentiated CMs in 3 different patches and the corresponding calcium upstroke and downstroke velocities. (**D**) Spatiotemporal calcium activity of a disk with 1 Hz pacing including isochronal maps of calcium response duration at 80% of repolarization (CaD80) and the activation time at 50% of depolarization (AT); red asterisks represent the site of pacing.

The optimized cell-laden bioink for printing cardiac disks is composed of three major components: GelMA/ColMA polymer precursor solution, an extracellular matrix (ECM) cocktail with fibronectin and laminin-111, and hiPSCs (Figure 3A(i)). Many studies have shown the importance of proper engagement between stem cells and ECM ligands for modulating cardiac differentiation [43,44,45]. Based on these, hiPSCs were first mixed with the ECM solution to ensure the uniform distribution and binding of cells and proteins. The cell solution was then combined with the precursor solution, and the resultant translucent ink was used immediately for printing. A 405 nm blue light was used for photo-crosslinking GelMA and ColMA during and after the FRESH printing process to ensure the disks had a defined structure (Figure 3A(ii)), and the mechanical properties of crosslinked bioink were similar to those of late embryonic hearts [37]. After crosslinking, the gelatin slurry bath was incubated at 37 °C to liberate the printed construct (Figure 3A(iii)). Printed disks were cultured for 13 days to allow for the formation of large cell colonies followed by the regulation of the Wnt/β-catenin pathway to drive cardiogenesis. After differentiation, cardiac disks were able to maintain the circular structure and displayed dense cell layers with clear contraction (Figure 3A(iv)).

#### 2.2.2. Bioprinted Cardiac Disks Exhibit Contiguous Calcium Activity

Bioprinted cardiac disks were cultured until the day of cryoablation. To examine the electrochemical dynamics of the cardiac disks, calcium transients were recorded from predetermined regions that corresponded to disk radii and, therefore, gave a uniform evaluation of the center and edge regions of each disk (Video S2, *n* = 3 disks, 3 regions per disk). The traces from different disks were similar to one other (Figure 3B), and regular calcium fluxes could be observed from all of the traces. The peak amplitudes did not decline with time, suggesting a stable calcium handling ability. The upstroke and downstroke velocities were also measured from the recorded traces (Figure 3C), and the calcium release rate (0.289 ± 0.102/s, *n* = 3) was higher than the uptake rate (0.093 ± 0.022/s, *n* = 3, *p* = 0.031), displaying the same trend as human cardiomyocytes [46].

To assess the electrochemical activities throughout the whole disk surface, optical mapping was used to visualize spatiotemporal calcium flux propagations at the macroscale. Cardiac disks were paced at 1 Hz, and the isochronal maps of calcium potential duration at 80% repolarization (CaD80) as well as activation time at 50% depolarization (AT) were generated from the recorded fields of view (Figure 3D). The average CaD80 was 684.8 ± 90.7 ms, and the detectable calcium signals covered over half of the disk surface (56.7%). The undetectable areas likely represent relatively unexcitable cells or dead areas. Calcium signal propagation was presented in the AT map where the pacing site behaved as one of the signal origins. Of note, the pacing site was not the only initiating area of calcium signal, suggesting the existence of cells with intrinsic automaticity such as immature cardiomyocytes or pacemaker cells. Another possible explanation is that remaining calcium signals travelling through the undetectable area induced the calcium fluxes on the opposite site of the detectable area. Overall, the large conductive area supports use of the cardiac disk to evaluate cell responses to a medical device at the macroscale.

### 2.3. Cryoablation on the Bionic Testbed

A typical cryoablation system is composed of a CryoConsole™ connected to a cryoablation catheter via cords for refrigerant delivery and electrical control [47]. The CryoConsole™ contains compressed refrigerant as the cooling source, and once the refrigerant is delivered to the catheter tip, it undergoes vaporization and volume expansion to obtain a reduced temperature. To perform the cryoablation process on the bionic testbed, a saline bath at 37 °C was used for maintaining the system temperature, and the cryoablation catheter was attached to an adjustable stage, enabling the modulation of the contact force. The operating temperature was set to −80 °C in the CryoConsole™ to ensure permanent tissue damage [48]. During the process, ice formation at the tip–tissue interface was clearly observed (Video S3), which not only created cryoablated lesions but also helped stabilize the tip via cryoadhesion [49].

#### 2.3.1. Correlated Pressure Profile and Cell Response to Cryoablation

The contact force at the catheter tip–tissue interface plays an important role in determining lesion size [50]. To examine the effects of the contact force during the cryoablation of cardiac disks, the bionic testbeds were treated with 0.1 N and 0.01 N contact forces for the normal therapy (high contact) and failed contact (low contact) conditions, respectively. A contact-force-only control group was also introduced by applying 0.1 N without cooling, and pressure maps were recorded for all three conditions.

**Figure 4:**
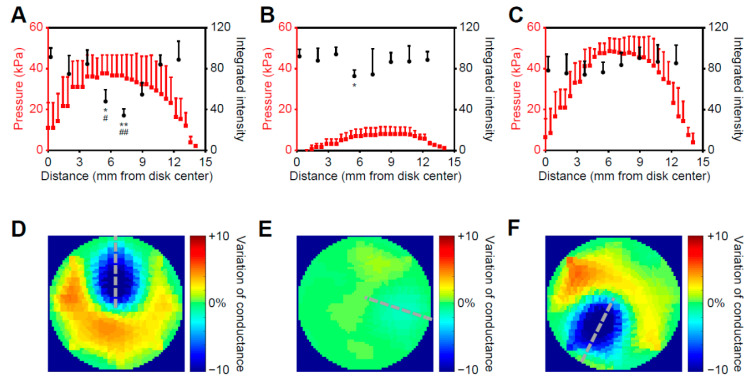
Calcium intensities after cryoablation and recorded pressure profiles along the radius of disks are presented in the same figure to show the correspondence. (**A**–**C**) represent the conditions of normal pressure with cryoablation, minimum pressure with cryoablation, and normal pressure without cryoablation, respectively. The red dots and the black dots indicate the recorded pressures and the calcium intensities of cardiomyocytes from the same radius of each disk, respectively. (**D**–**F**) are reconstructed conductance maps showing three pressing locations under normal pressure with cryoablation, minimum pressure with cryoablation, and normal pressure without cryoablation, respectively. The gray-dotted lines indicate the pressure profiles in figure (**A**–**C**). * *p* < 0.05, ** *p* < 0.01 for comparison between the pre-ablation group and the post-ablation group (*n* = 3), two-tailed Student’s *t*-test. # *p* < 0.05, ## *p* < 0.01 for comparing to the group nearest to the disk center (0.2 mm) (*n* = 3), 1-way ANOVA with Tukey post hoc test.

After cryoablation, cardiac disks were detached from the pressure sensor and stained again with a calcium-sensitive dye for post-ablation imaging. Low- or no-intensity areas indicative of a lack of calcium transients were observed around the ablated area for the normal therapy condition group, and the catheter location (7.5 mm from disk center) properly corresponded to the area with the lowest calcium intensity (7.2 mm from disk center) (Figure 4A, black dots). The calcium intensity was significantly decreased compared to both the same area before ablation (Figure S6A and Figure 4A, black dots) and the relatively unaffected area nearest to the disk center (0.2 mm), indicating that cryoablation did cause tissue damage to the testbed area surrounding the catheter tip. Calcium intensities were only slightly decreased for the low/failed-contact group (Figure S6B and Figure 4B, black dots), and only the calcium intensity in the area 5.5 mm from the disk center was significantly decreased after cryoablation. There was no significant calcium intensity decline for the high-contact (no cryoablation) group (Figure S6C and Figure 4C, black dots), suggesting the normal contact force for therapy (0.1 N) induces no remarkable damage to human cardiac tissue.

To verify if the applied pressure was spatially reflected in the cell response, pressure maps recorded for each condition (Figure 4D–F) were used for generating pressure profiles along the radii of disks across the ablated regions (Figure 4A–C, red dots; and Figure 4D–F, gray-dotted lines). For the normal therapy condition (Figure 4A), a negative correlation was observed between the calcium intensity and applied pressure (Pearson’s correlation of the nearest data pairs, ρ = −0.719, Figure S7). For the failed/low-contact condition, a weaker negative correlation between calcium intensity and applied pressure was observed (Figure 4B, ρ = −0.616). The cell response and the applied pressure profile were unrelated in the force-only group (Figure 4C, ρ = 0.193). Overall, the significant correspondence between the cell response and the applied pressure suggests that this bionic testbed can be useful for defining the cryoablation area and evaluating the outcomes of tissue and cell damage.

#### 2.3.2. Post-Ablation Cardiac Disks Show Tissue- and Cell-Scale Damage

**Figure 5:**
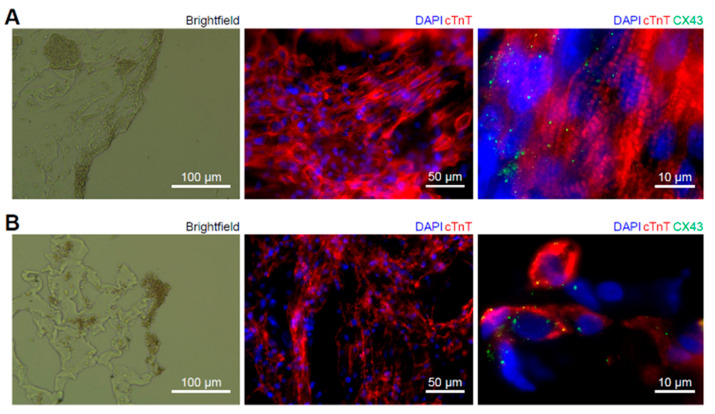
Cell and tissue responses to the cryoablation. (**A**) From left to right, the non-ablated disk under brightfield, staining for cardiac muscle layer via cTnT (cardiac troponin T) and DAPI on the surface of the non-ablated disk, and a high-magnification image of cell structures of the non-ablated disk stained via cTnT, DAPI and CX43 (connexin 43). (**B**) From left to right, the ablated disk under brightfield, staining for cardiac muscle layer via cTnT (cardiac troponin T) and DAPI on the surface of the ablated disk, and a high-magnification image of cell structures of the ablated disk stained via cTnT, DAPI and CX43.

Cardiac disks were washed and fixed for immunofluorescence staining to reveal structural changes after cryoablation. The intact bioink structure and the contiguous cell layer can be observed from the brightfield cross-sectional view of the non-ablated area (Figure 5A, left). For the ablated area, although the crosslinked bioink was not fully degraded, there were numerous cavities and disconnected cell colonies, likely due to the formation of extracellular ice crystals at the beginning of cryoablation (Figure 5B, left). This led to the movement of water out from cells and caused intracellular desiccation [51]. As a result, disarrayed sarcomeric proteins (cTnT) were found from the top view of the ablated area (Figure 5B, middle), while the non-ablated area exhibited an appropriately organized cTnT network (Figure 5A, middle). The cell morphology changed after ablation was further confirmed by the decreased fiber length of cTnT proteins (non-ablated disk: 14.717 ± 2.237 µm vs. ablated disk: 9.472 ± 1.362 µm, *p* < 0.01). To determine the extent to which cell–cell connectivity was preserved with cryoablation, cardiac disks were cryosectioned and stained for gap junction protein connexin 43 (CX43). A strong and uniform expression of CX43 was presented in cardiac disks without cryoablation, as well as clear sarcomeric striations with cTnT staining (Figure 5A, right). On the other hand, CX43 was mostly distributed around shrunk cell nuclei with completely disrupted and collapsed cTnT expression in cryoablated tissues (Figure 5B, right), suggesting that the cryoablation procedure performed in this study did cause tissue and cell damage to the bioprinted human cardiac disks. However, many cells survived despite the disrupted state.

## 3. Discussion

As technology advances, engineered functional cardiac tissues are being created as model systems for in vitro drug screening [52,53] and as future therapeutic grafts. These types of tissues have also been proposed as a means to test the performance of medical devices and the impact of devices on tissue health and function. However, a human tissue-based testbed for clinical-grade medical devices remains an unmet need in the field. While animal models and tissue phantoms have been used to show the impact of ablation catheters on the surrounding tissue area [54,55], limitations such as sample variations and differences in human tissues restrict their utility. In addition, existing systems lack the capability to monitor environmental conditions at the time of and after device intervention. Our goal was to develop centimeter-scale human myocardium constructs integrated with sensing elements to determine the impact of applied force with cardiac ablation on the viability and function of human cardiac muscle cells.

Here, we developed a bionic myocardial testbed through interdisciplinary expertise: stem cell proliferation and differentiation in a 3D-printed ECM, and flexible hydrogel-based electronic sensors. The ECM-based bioink developed in our previous study [37] was printed into centimeter-scale disks with hiPSCs. After the full differentiation of hiPSCs to cardiomyocytes, the disks were attached to an EIT sensor which contained an ionic hydrogel as the conductive material. This interdisciplinary approach exhibited tissue-like stiffness and responsiveness for detecting different levels of strain [34]. Verification of the sensor demonstrated a linear pressure–conductance relationship within the range of normal contact force during cryoablation (Figure 2C). However, one limitation of this approach is that the electrical conductance of the hydrogel also depends on the temperature. For this reason, we maintained a consistent temperature for the duration of the test. Going forward, further optimization of the hydrogel composition may enable multimodal sensing to map pressure and temperature simultaneously.

In most cardiac ablation testing systems, a bulky liquid phase is introduced to replicate the body environment in vitro [56]. Here, we performed cryoablation procedures in a 37 °C water bath with PBS to mimic the blood phase after the system reached thermal equilibrium. Ice crystals formed and grew when cryoablation procedures were applied to the testbed (Video S3). This allowed the catheter tip to adhere to the tissue surface, which is typically regarded as the reason for the phenomenon that cryoablation exhibits a more defined lesion area compared to other ablation methods [49]. The water bath provided a temperature-stable environment. In future studies, circulating pumps or extra pipes can be introduced to the system to further replicate physiological fluid dynamics [57].

Another important factor affecting the outcome of cardiac ablation is catheter contact force. We verified the correspondence between the tissue responses and pressure readings to cryoablation by plotting the pressure and post-ablation calcium intensity profiles together (Figure 4). While the pressure readings were similar for runs with and without cryo application, only the group treated with cryoablation showed a negative correlation where the highest pressure and the lowest calcium intensity occurred around the catheter center (7.5 mm from disk center). The group with low contact force exhibited minimal cell responses to cryoablation. Our results demonstrated that the bionic myocardial testbed can not only provide the spatial information of applied pressure and cell response but can also help predict the outcome of conditions with different contact forces. However, since the maximum thickness of the tissue from our current fabricating process is 0.5 mm [37], it is notable that the cardiac disks did not have sufficient thicknesses to provide post-ablation outcomes for a depth map. Strategies such as the stacking of engineered cardiac tissues [58] or introducing dynamic culture via bioreactors could be further explored to provide information regarding the ablated region depth. Given that 3D printing was used in the fabrication of both sensors and tissues, it is possible to expand this simple testbed to a more complex tissue–sensor assembly to better replicate the anatomical geometry of the region of interest. This will require advanced manufacturing methods and materials to fabricate all-in-one tissue–sensor composites and incorporate surrounding tissues.

Finally, the cryoablation impact on hiPSC-derived cardiomyocytes was further examined by immunofluorescence staining (Figure 5). Structure disruption was confirmed by damaged bioink and fragmented cTnT expression. In addition, cell damage in the ablated area was identified via diminished cell nuclei with condensed cTnT and gap junction CX43 expression, indicating that the cryoablation applied to testbeds did indeed interrupt cell–cell connectivity between cardiomyocytes. The results suggest that recurrent arrhythmia, as observed in many patients receiving catheter ablation [15,18], could occur as a function of altered sarcomeric structure and disrupted cell–cell connectivity. Further mechanistic details could be evaluated using RNAseq or other omics approaches to identify targets to promote cell health or remove compromised cardiomyocytes.

Future studies should also consider conducting a long-term culture of ablated tissues for investigating the recurrence rate under different conditions, as well as to determine how signal transduction resumes among cells. A fully sterilized ex vivo ablation system would be useful to prevent contamination and allow the observation of long-term post-ablation events. Optical mapping and immunostaining could then be used for examining the resumption of electrical activity and cell connectivity, respectively. The mechanisms involved in tissue recovery and rhythm recurrence could be further studied using techniques such as RNA-seq. This should accelerate the development of more efficient therapies for cardiac arrhythmias.

In this study, 3D-bioprinted cardiac disks were integrated with soft-pressure sensors to serve as a testbed to evaluate cryoablation procedures as a means of studying cell behaviors following ablation. In the future, this platform could be applied not only for radiofrequency ablation, where contact pressure plays a key factor in the safety and the effectiveness of the procedure, but also for investigating the cell selectivity of advanced ablation techniques such as pulse field ablation. In this case, cardiac disks composed of multiple cardiac cell types (e.g., cardiomyocytes, endothelial cells, smooth muscle cells, fibroblasts) could be employed. In addition to cardiac ablation, bionic myocardial testbeds of this type will be valuable for evaluating many different cardiac device interventions (e.g., cardiac pacemakers), in which outcomes inform surgical procedures and drive the discovery of human tissue dynamics during and after intervention.

## 4. Materials and Methods

### 4.1. Material System for the Pressure Sensor

The ionic hydrogel consisted of 10.45 wt.% acrylamide (Sigma-Aldrich, Inc., St. Louis, MO, USA) as the monomer, 22.66% wt. lithium chloride (Sigma-Aldrich, Inc., St. Louis, MO, USA) for ion induction, 0.007 wt.% N,N′-Methylenebisacrylamide (Sigma-Aldrich, Inc., St. Louis, MO, USA) as the crosslinking agent, 0.06 wt.% 2-Hydroxy-2-methylpropiophenone (Sigma-Aldrich, Inc., St. Louis, MO, USA) as the photoinitiator, and 66.82 wt.% ultra-pure water as the solvent. After thorough mixing, the resulting hydrogel precursor was cast in the sensor mold and crosslinked via exposure to UV light (wavelength 320–500 nm, OmniCure Model S1500, Excelitas Technologies Corp., Waltham, MA, USA). The silicone material was prepared by mixing parts A and B of Ecoflex 00-30 (Smooth-On, Inc., Macungie, PA, USA) with a ratio of 1:1. The mixture was crosslinked at room temperature for four hours to form the silicone encapsulation.

### 4.2. Compression Test

The cylindrical samples of ionic hydrogel and silicone rubber (15 mm diameter, 8 mm height) were prepared by casting the materials in 3D-printed plastic molds. Static compression tests were performed using a mechanical analyzer (RSA-G2, TA Instruments, Inc., New Castle, DE, USA). The static compression tests were conducted at a rate of 0.02 mm/s for a duration of 100 to 200 s.

### 4.3. Periphery Circuitry Design for the Pressure Sensor

The operating circuit consisted of a microcontroller (Teensy 3.6, PJRC.COM, LLC, Sherwood, OR, USA) for impedance measurement and data acquisition, a waveform generator (RIGOL DG1022, RIGOL Technologies, Portland, OR, USA) and a voltage-controlled current source (LT6375, Analog Devices Inc., Wilmington, MA, USA) as a power supply, an ADC pre-amplifier circuit (INA128P, INA111AP, Texas Instruments, Dallas, TX, USA) for noise filtering and signal amplification, and input/output multiplexers (CD74HC4067, Texas Instruments, Dallas, TX, USA) for switching electrodes for current sourcing and voltage measurement. The AC signal supplied to the pressure sensor was 0.8 mA at a frequency of 20 kHz.

### 4.4. Software Interface for the Pressure Sensor

The sensor design with eight electrodes allowed 40 voltage measurements (with adjacent stimulation and measurement patterns) per estimation of the pressure map. Within each voltage measurement, the microcontroller was programmed to first sample 100 voltage values at a frequency of 400 kHz (covering approximately 5 periods of the 20 kHz AC signal), and then perform root mean square (RMS) estimation of the voltage amplitude. The data package consisting of the RMS values of the 40 voltage measurements was sent to a PC at a refresh rate of 2.5 Hz for real-time pressure estimation. The PC software was developed based on the EIDORS toolkit in MATLAB (MathWorks, Natick, MA, USA). The conductance map was reconstructed based on the one-step Gauss–Newton inverse model with a NOSER prior. The conductance map was then scaled to the pressure distribution based on the linear model assumption and the calibrated ratio.

### 4.5. Sensor Calibration

Each conductance map generated by the EIT software was discretized into 576 triangular elements, each of which represented an area of ~1.23 mm^2^ within the circular sensing region. To estimate the pressing location, a region consisting of six elements with the lowest conductance was first located in the conductance map (Figure 2E), with the overall area similar to that of the presser (3 mm diameter). Next, the pressing location was estimated as the centroid of the specified region. To characterize the spatial sensing error, defined as the difference between the actual and the intended pressing locations, a 2D rigid body transformation was computed by utilizing the iterative closest point (ICP) method in order to transform the actual pressing locations to the coordinate system where the intended pressing locations were defined (executed by the gantry system).

### 4.6. Cell Culture and Bioink Preparation

The hiPSC line was derived from cardiac fibroblasts and kindly provided by Dr. Jianyi Zhang (University of Alabama at Birmingham, Birmingham, UK). The cells were mycoplasma-free and were maintained in mTesR™1 and passaged with ReLeSR™ every 3–5 days. Only cells with passages <70 were used in this study. To prepare for the bioprinting of centimeter-scale cardiac tissues, an ECM-based bioink optimized for hiPSC proliferation and cardiac differentiation was used as previously described. hiPSCs were dissociated for 8 min with Accutase^®^ (cat#AT-104, Innovative Cell Technologies, Inc., San Diego, CA, USA) and resuspended in mTesR™1 with 187.5 μg/mL fibronectin (FN, cat# 356008, Corning Life Science, Tewksbury, MA, USA), 187.5 μg/mL laminin (LN, cat# 354259, Corning Life Science, Tewksbury, MA, USA), and 10 μM ROCK inhibitor to a concentration of 30 million cells/mL. The cell suspension was subsequently mixed 1:1 with gelatin methacrylate (GelMA, kindly provided by the Bioprinting Facility, UMN)/collagen methacrylate (ColMA, cat#5198, Advanced BioMatrix, Carlsbad, CA, USA)/lithium phenyl-2,4,6-trimethylbenzoylphosphinate (LAP, cat#900889, Sigma-Aldrich, St. Louis, MO) precursor solution to produce the final cell-laden bioink containing 15 million cells/mL with 10% GelMA, 0.25% ColMA, 93.75 μg/mL LN and FN, 0.5% LAP, and 5 μM ROCK inhibitor.

### 4.7. Bioprinting and Differentiation of Human Cardiac Disks

To create human cardiac tissues as catheter ablation testbeds, a simple disk model 3 cm in diameter and 1 mm in thickness was generated for bioprinting. For printing with the low-viscosity bioink, the Freeform Reversible Embedding of Suspended Hydrogels (FRESH) method was applied as previously described. Cardiac disks were printed on an INKREDIBLE+ Bioprinter (CellInk, Gothenburg, Sweden) into the FRESH support bath maintained at room temperature. Pneumatic pressure was set to achieve optimal ink flow from the needle maintained at 27 °C by a customized heating jacket, and was within the range of 25–35 kPa. After printing, the disks were crosslinked with a 405 nm flashlight for 20 s on both sides, and then the temperature of the container was raised to 37 °C for 30 min to completely dissolve the support bath. The printed disks were then rinsed three times with PBS before being transferred to a T25 flask with 20 mL mTeSR^TM^1 media and 5 μM ROCK inhibitor.

Then, 3D-printed disks were cultured in mTeSR^TM^1 media for 13 days, beginning the day after printing, with daily medium changing for cells to proliferate. Cardiac differentiation was performed according to the previously published method [37] and was initiated 2 weeks after printing by changing the culture media to RPMI + B-27 Supplement minus insulin (cat#A1895601, Gibco, Waltham, MA, USA) supplemented with 12 μM CHIR99021(cat#SML1046, Sigma-Aldrich, St. Louis, MO, USA). After the disks were treated with CHIR for 24 h, media were removed and replenished with fresh RPMI + B-27 minus insulin media. On Day 3 of differentiation, 5 μM IWP-2 (cat#3533, Tocris Bioscience, Minneapolis, MN, USA) was added in half old/half fresh RPMI + B-27 minus insulin media for the culturing disks. On Day 5 and Day 7, the RPMI + B-27 supplement with insulin (cat#17504044, Gibco, Waltham, MA, USA) was used for medium changes, and this was repeated every three days after that. Starting on Day 20 of differentiation, cardiomyocyte enrichment was performed by treating the disks with glucose-free DMEM containing 4 mM sodium L-lactate (cat#L7022, Sigma-Aldrich, St. Louis, MO, USA) for 4 days total, with fresh lactate media replenished on Day 22. On Day 24, disks were recovered by washing with PBS and replacing the media with RPMI + B-27 with insulin. After that, medium changes were performed every 3 days until the end-point test. It should be noted that the cardiomyocytes generated here were not fully characterized for chamber specificity, and cell-sorting or differentiation protocols specific to atrial cells can be developed and implemented in the future. Cryoablation was performed on disks at ≥6 weeks after fabrication. Calcium and optical mapping assessments were performed both right before and after the cryoablation, and the disks were then fixed for staining.

### 4.8. Calcium Transient Measurement

Calcium movement and intensity within the cardiac disks were measured on the same day of cryoablation. Both calcium activities before and after the cryoablation were recorded by a DMi8 fluorescence microscope (Leica, Wetzlar, Germany). RPMI + B-27 supplement with insulin with 5 µM Rhod-2 acetoxymethyl ester (Rhod-2 AM, cat#50024, Biotium, Fremont, CA) was first used for incubating the cardiac disks at 37 °C. After 30 min, the dye solution was removed and replenished with Tyrode’s salt solution for another 30 min incubation. Imaging was performed on the microscope stage with a heating plate to ensure the temperature was at 37 °C during the process. At least three videos were recorded from each disk at randomly selected areas to generate calcium traces, which are shown in normalized intensity (F/F_0_) versus time, along with the average upstroke and downstroke velocities. Images were taken along the radius of each disk at regular intervals before cryoablation and across the ablated regions along the same radius after cryoablation to show the treatment effects on cardiac disks. Integrated calcium intensities were acquired from each whole image using Fiji (ImageJ; National Institutes of Health, Bethesda, MD, USA) and normalized to the highest value of each disk (F/F_max_).

### 4.9. Optical Mapping Assessment

The optical mapping of the cardiac disks was performed based on previously published work [59], except the dye was changed to Rhod-2 AM and the incubation process was modified to allow the calcium transient measurement and optical mapping to be simultaneously accomplished on the day of cryoablation. Briefly, after calcium transient measurement, cardiac disks were transferred to 10 cm dishes with 5 µM Rhod-2 AM in 20 mL 1/1 Krebs-Ringer Buffer/ RPMI + B-27 supplement with insulin. After incubation for 15 min, a two-diode-pumped continuous-excitation green laser (581 nm, 1 W; Shanghai Dream Lasers Technology, Shanghai, China) was used, and fluorescence intensity was recorded for 15 s using 14-bit, 80 × 80-pixel resolution cameras (Little Joe, RedShirt Imaging, SciMeasure, Decatur, GA, USA) at 500 frames per second. Pacing was provided at 1 Hz via a bipolar electrode and optical mapping movies were recorded during pacing. Optical calcium duration (CaD) was measured at 80% repolarization, and two-dimensional (2D) CaD maps were constructed to reveal the spatial distribution of CaD on the structure surface.

### 4.10. Cryoablation of Bionic Myocardial Testbed

After optical mapping, cardiac disks were attached to the center of the hydrogel-based soft-pressure sensor. Then, 100 µL bioink was added to the edge of the disk and crosslinked to stabilize the structure. The testbed was then transferred to a 37 °C water bath containing sterile phosphate-buffered saline solution (PBS), and the ablation catheter (Freezor MAX, Medtronic, Minneapolis, MN, USA) was applied to the cardiac disk surface at a location 7.5 mm away from the disk center. The contact force was calibrated by the pressure sensor and set at either 0.1 N as the normal therapy condition or 0.01 N as the failed contact condition. Ablation duration was set via the CryoConsole™ to 60 s. A group with 0.1 N contact force was treated with the same process without turning on the CryoConsole™ as a contact-force-only control. After cryoablation, cardiac disks were gently detached from the pressure sensor by a stainless-steel spatula and used for the post-ablation calcium assessment.

### 4.11. Immunohistological Assessment

After post-ablation imaging, cardiac disks were fixed in 4% PFA for 24 h. Samples for cryosection were treated with 30% sucrose for 24 h prior to OCT embedding and then sectioned at a thickness of 5-7 µm. Before staining, samples were incubated with 0.2% Triton-X-100 for an hour followed by another 2 h incubation with the blocking buffer containing 5% BSA, 0.1% Triton X-100, 2% goat serum, and 1% glycine. Cardiac troponin T (cTnT) was stained by the primary antibody mouse anti-cTnT (1:200 dilution, cat#MS-295-P1, ThermoFisher, Waltham, MA, USA) and the corresponding secondary antibody goat anti-mouse AlexaFluor647 (1:500 dilution, cat#A21236, ThermoFisher, Waltham, MA, USA). Cell nuclei were stained with DAPI. Sectioned samples were also stained for connexin 43 (CX43) using rabbit anti-Cx43 (1:100 dilution, cat#ab11370, Abcam, Cambridge, UK) and goat anti-rabbit AlexaFluor488 (1:500 dilution, cat#A11008, ThermoFisher, Waltham, MA, USA). Samples were examined using a DMi8 fluorescence microscope (Leica, Wetzlar, Germany). To determine the fiber length of cTnT proteins, five 20× images were obtained from each condition and analyzed by using the Tubeness plugin in Fiji (ImageJ; National Institutes of Health, Bethesda, MD, USA) to identify fiber structures first and then using the Anamorf plugin [60] in Fiji to measure fiber length.

### 4.12. Statistical Analysis

Statistical analyses were conducted by Graphpad Instat3 (GraphPad Software, San Diego, CA, USA). Two tailed Student’s *t*-tests were used to compare the results between two individual groups, and one-way ANOVA with Tukey post hoc testing was used for experiments with >2 experimental groups. A *p*-value below 0.05 was considered significant.

## Data Availability

The authors declare that all data supporting the findings are presented within the article or are available from the authors upon request.

## Supplementary Materials

The following supporting information can be downloaded at: https://www.mdpi.com/article/10.3390/ijms232214444/s1.
