# Supplementary material for: A Bionic Testbed for Cardiac Ablation Tools
Source: Int J Mol Sci. 2022 Nov 21;23(22):14444. doi: 10.3390/ijms232214444 (PMC9692733; doi:10.3390/ijms232214444)
Supplement: Supplementary file 1 [file ijms-23-14444-s001.zip › ijms-1922260-supplementary.pdf]

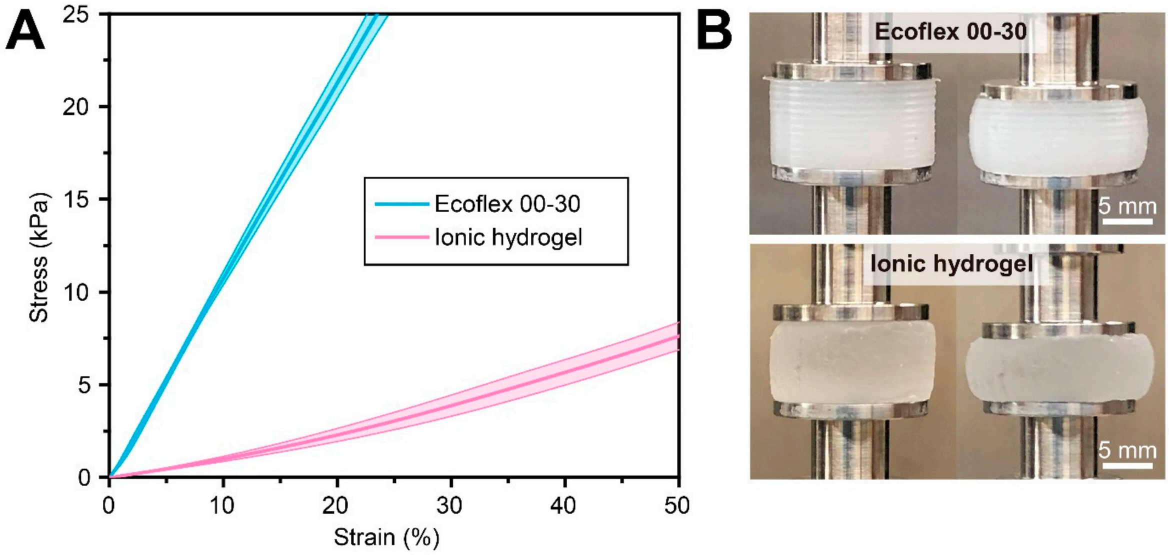

Figure S1. Compression test of silicone rubber and ionic hydrogel. (A) Strain-stress curve of the samples of silicone rubber and ionic hydrogel under compression (n = 4). (B) Photographs showing cylindrical samples of silicone rubber (top) and ionic hydrogel (bottom) during compression test.

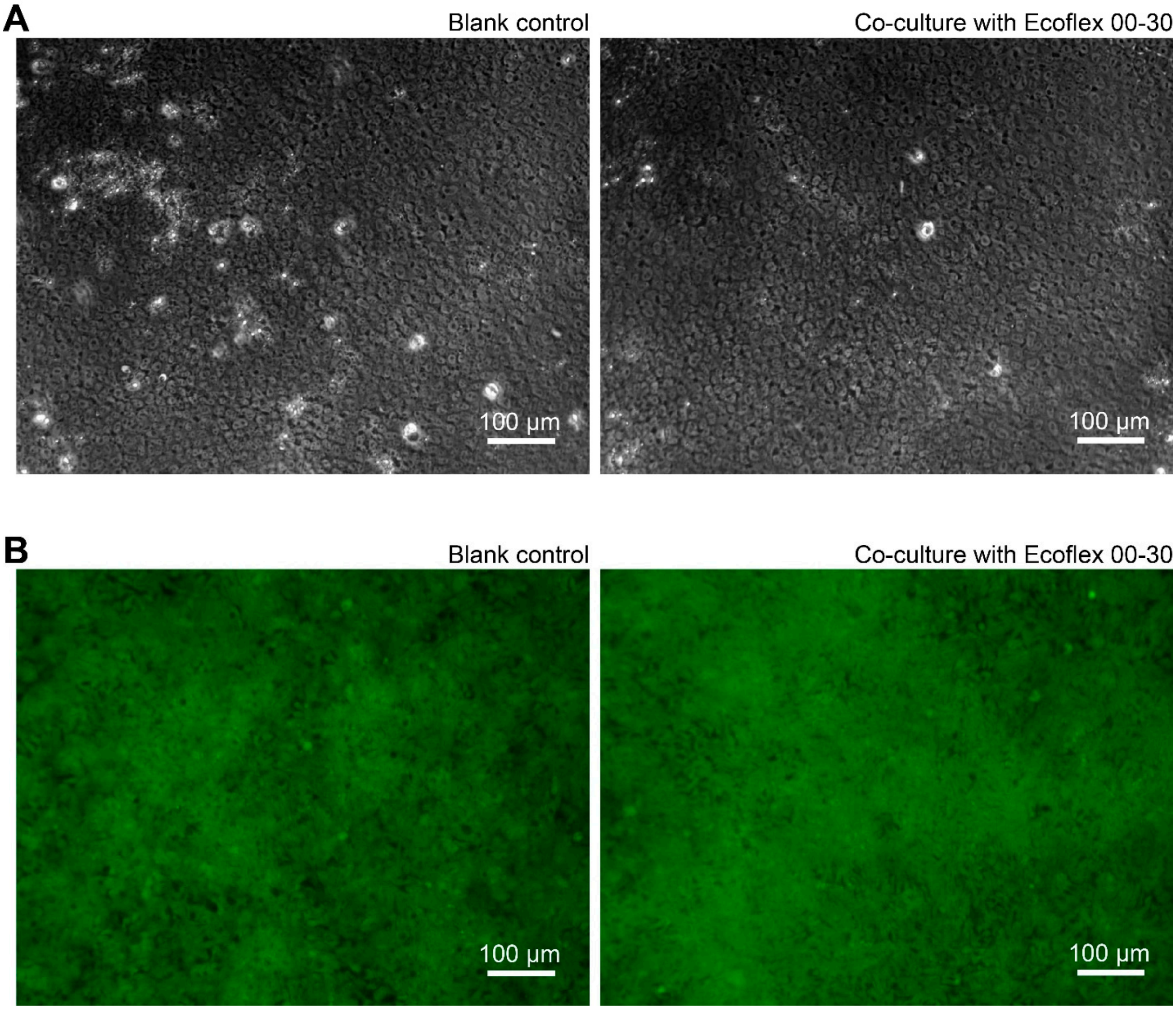

Figure S2 Biocompatibility of the sensor material, Ecoflex 00-30, to hiPSCs. (A) Brightfield images of cells after being cultured for 5 days. (B) Cells stained via LIVE/DEAD cytotoxicity kit after being cultured for 5 days. Blank control indicates the cells under normal maintenance.

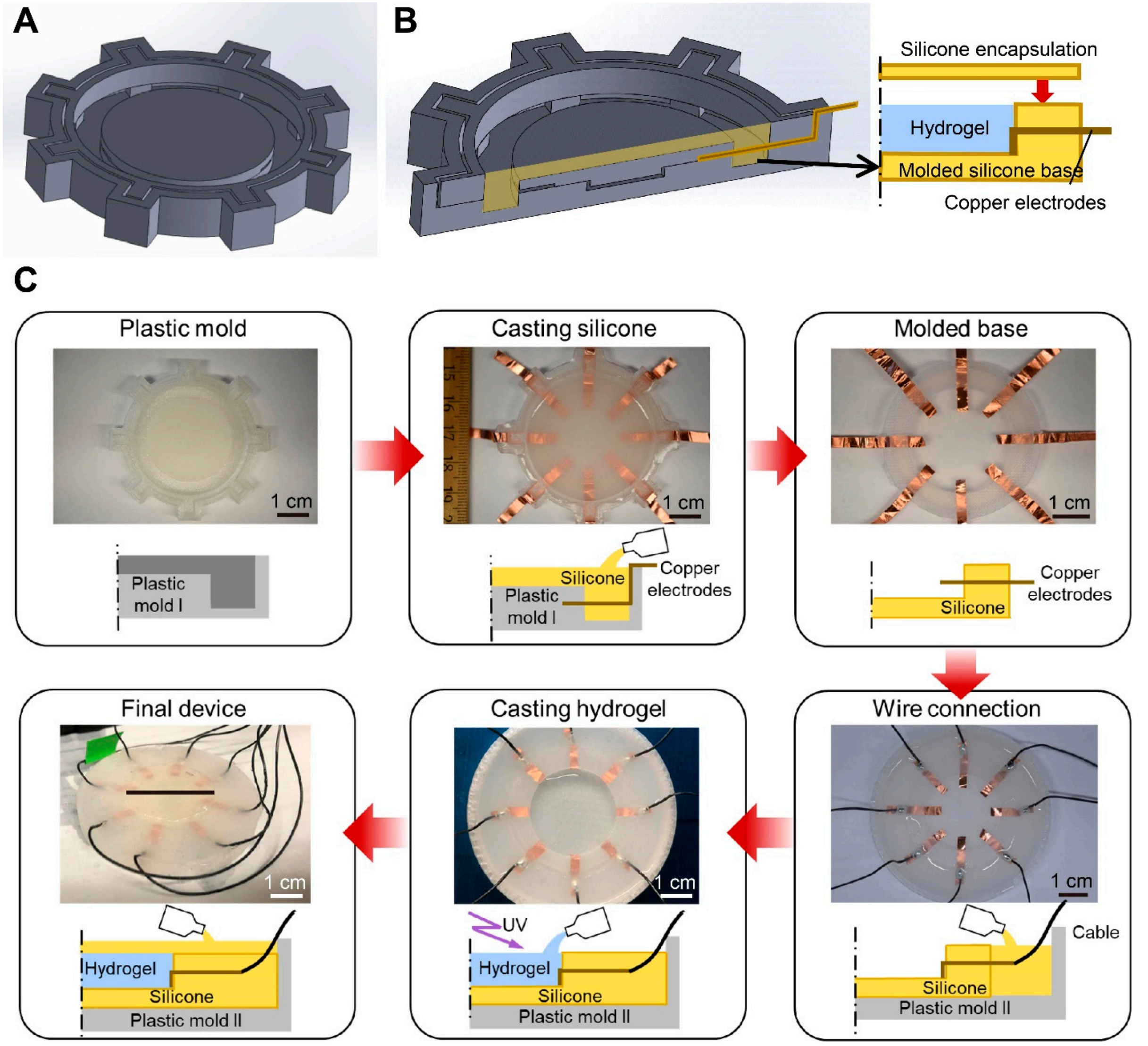

Figure S3. Fabrication process of the hydrogel-based EIT pressure sensor. (A) Schematic image of the 3D printed plastic mold assembly to embed the copper electrodes and cast the silicone substrate. (B) Cross-section view of the 3D printed mold assembly, with the yellow region showing the silicone substrate and the brown line showing the copper tape. (C) Photographs and schematic images showing multiple steps of the fabrication process.

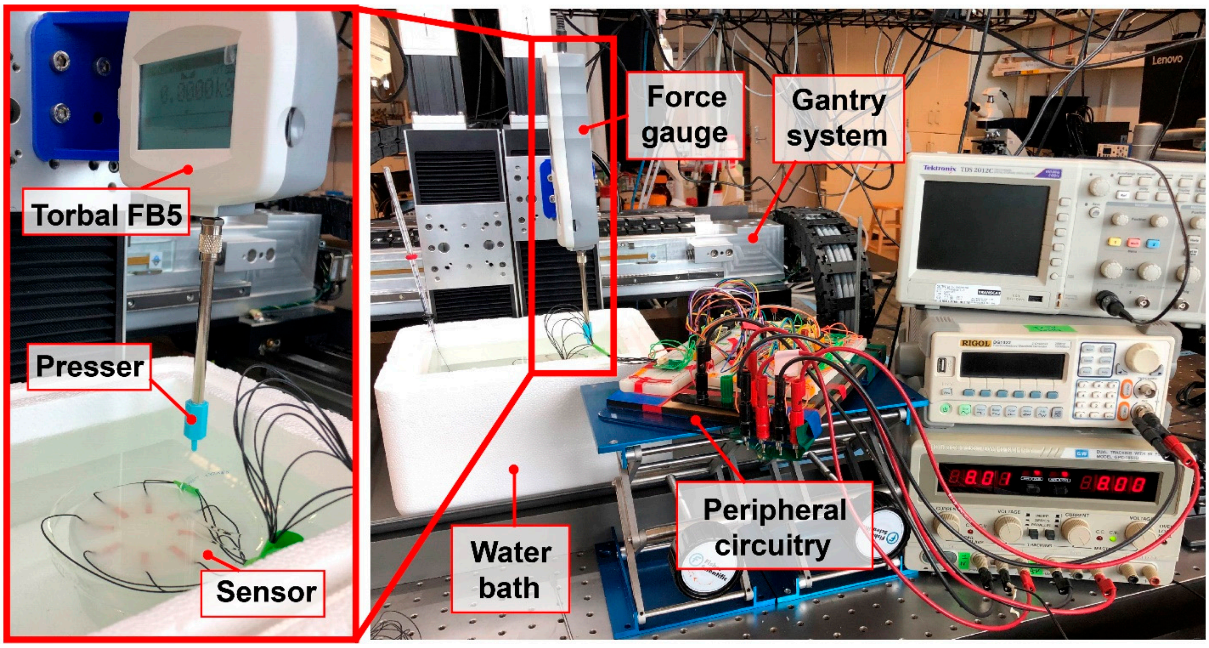

Figure S4. Photographs of the experimental setup for electromechanical characterization.

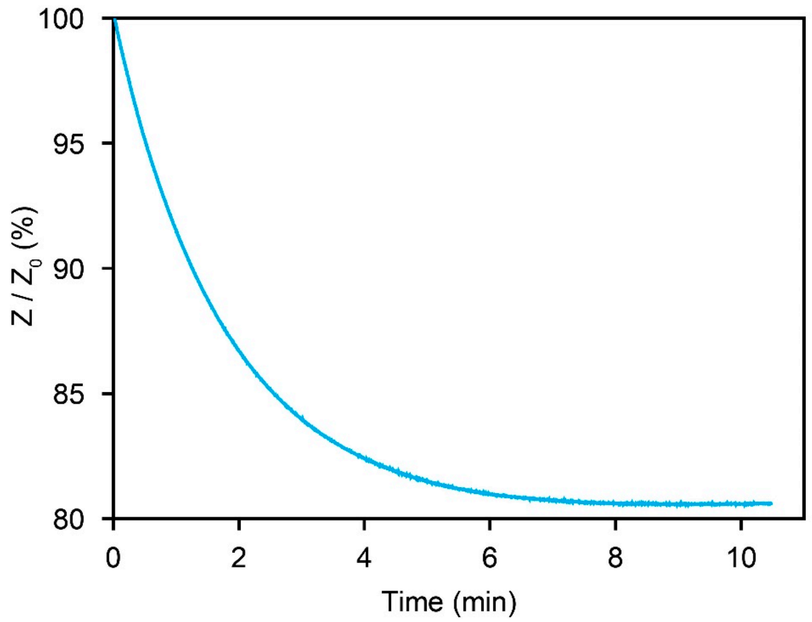

Figure S5. Plot showing the time response of Z/Z_0 of the pressure sensor after being placed in 35 °C water bath from air under room temperature. Z denotes current impedance. Z_0 denotes original impedance at time zero (in air). Each data point was averaged over 40 EIT measurements from the electrodes.

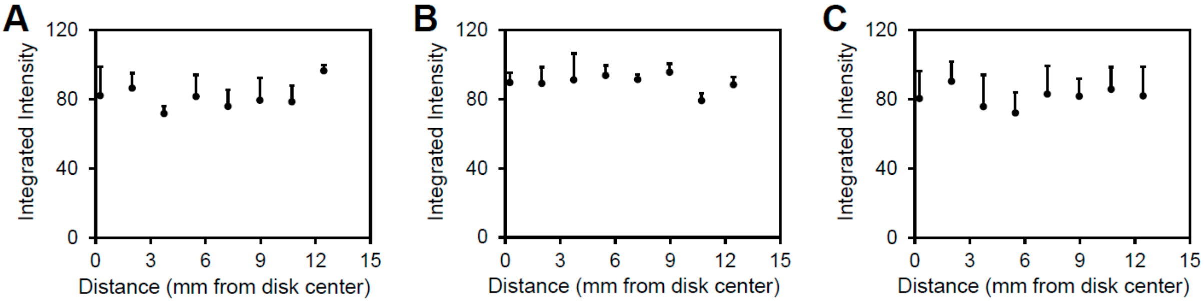

Figure S6. Calcium intensities before cryo-ablation along the same radius of disks where post-ablation measurements were conducted. (A), (B), and (C) represent the groups of disks before testing under conditions of normal pressure with cryoablation, minimum pressure with cryoablation, and normal pressure without cryoablation, respectively.

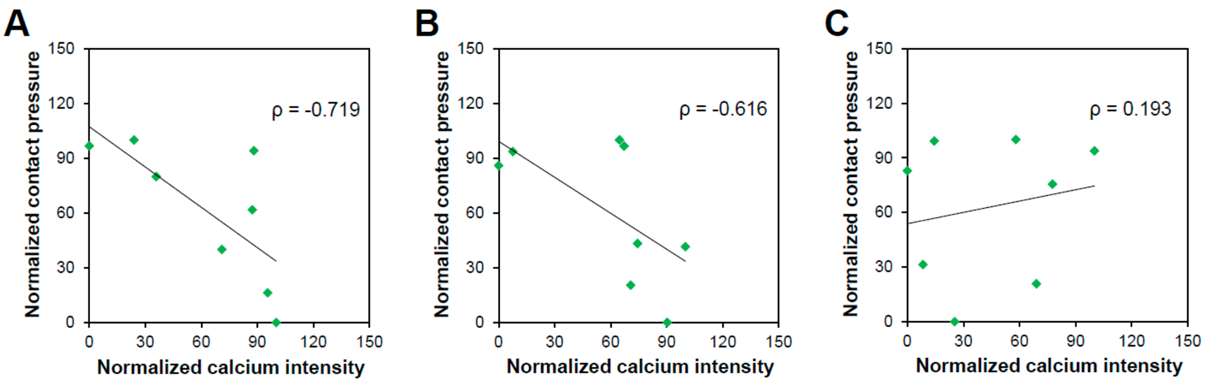

Figure S7. Normalized nearest pressure-intensity pairs from Figure 4 with corresponding Pearson's correlation coefficients. (A), (B), and (C) represent the conditions of normal pressure with cryoablation, minimum pressure with cryoablation, and normal pressure without cryoablation, respectively.

Videos are in real-time unless otherwise specified.

Video 1 Real-time response of the pressure sensor

Video 2 Calcium activity of the cardiac disk before ablation

Video 3 The cryoablation process
